# Facile template-free synthesis of pine needle-like Pd micro/nano-leaves and their associated electro-catalytic activities toward oxidation of formic acid

**DOI:** 10.1186/1556-276X-6-381

**Published:** 2011-05-13

**Authors:** Rong Zhou, Weiqiang Zhou, Hongmei Zhang, Yukou Du, Ping Yang, Chuanyi Wang, Jingkun Xu

**Affiliations:** 1 College of Chemistry, Chemical Engineering and Materials Science, Soochow University, Suzhou, 215123, People's Republic of China; 2 Xinjiang Technical Institute of Physics & Chemistry, Chinese Academy of Sciences, Urumqi, 830011, People's Republic of China; 3 Jiangxi Key Laboratory of Organic Chemistry, Jiangxi Science and Technology Normal University, Nanchang, 330013, People's Republic of China

## Abstract

Pine needle-like Pd micro/nano-leaves have been synthesized by a facile, template-free electrochemical method. As-synthesized Pd micro/nano-leaves were directly electrodeposited on an indium tin oxide substrate in the presence of 1.0 mM H_2_PdCl_4 _+ 0.33 M H_3_PO_4_. The formation processes of Pd micro/nano-leaves were revealed by scanning electron microscope, and further characterized by X-ray diffraction and electrochemical analysis. Compared to conventional Pd nanoparticles, as-prepared Pd micro/nano-leaves exhibit superior electrocatalytic activities for the formic acid oxidation.

## Introduction

Energy storage devices including fuel cell, Li-batteries etc. have been developing especially today [1,2]. Direct formic acid fuel cell has been receiving much attention as one of the most attractive energy sources [3]. Palladium (Pd) was found to show superior catalytic activity for formic acid electrooxidation compared with Pt-based catalysts [4,5]. Considerable efforts have currently been directed to developing novel Pd catalysts. Due to high-surface area and other unique physicochemical properties, nano-catalysts are known to have a significant effect on promoting the electro-oxidation of formic acid. Well-controlled nanostructures are thereby essential for achieving high efficient catalysts used in fuel cells. From this prospect, Pd nanoparticles with a variety of shapes have been explored, such as microspheres [6], polygonal nanoparticles [7,8], nanotubes [9], nanothorns [10], nanorods [11], and nanowires [12-15]. Sun et al. reported the efficiency of formic acid electro-oxidation can be improved by changing the morphology of the Pd nanostructures from nanoparticle to nanowire [16].

Recently, much attention has been paid to the synthesis of nanomaterials on the basis of electrochemical deposition methods because of their simple operation, high purity, uniform deposits, and easy control [17-19]. In order to obtain nano-architectural Pd catalysts directly grown on substrates by electrodeposition, templates are commonly used [20]. However, the fabrication is relatively complicated with multiple steps. Recently, a few studies on nano-architectural Pd fabrication using direct template-free electrodeposition on an indium tin oxide (ITO) electrode have been reported [21,22]. Park et al. reported the potentiostatic electrodeposition of Pd dendritic nanowires on an ITO electrode in a solution containing 0.2 M H_3_BO_3 _and 0.2 M PdSO_4 _[21], and they did not find the formation of Pd dendritic nanowires on the ITO substrate through potentiostatic reduction of PdCl_2_. Kwak et al. reported the electrodeposition of Pd nanoparticles on an ITO electrode by a cyclic voltammetry method in a 0.1 M H_2_SO_4 _+ 0.1 mM PdCl_2 _+ 0.2 mM HCl solution and their catalytic properties for formic acid oxidation [22]. Clearly, the composition of electrolytes and the different electrochemical methods employed for electrodeposition are critical to the morphology of the formed metal products. The present article provides a facile, one-step, template-free electrodeposition route of Pd micro/nano-leaves. As-formed Pd micro/nano-leaves were found to show promising activity for formic acid electro-oxidation.

## Experimental

### Materials and apparatus

PdCl_2 _(Shanghai Sinopharm Chemicals Reagent Co., Ltd., China) was used as received. Formic acid, H_3_PO_4_, and H_2_SO_4 _were of analytical-grade purity. Doubly distilled water was used throughout. A 1.0 mM H_2_PdCl_4 _solution was prepared by dissolving 0.1773 g of PdCl_2 _in 10 mL of 0.2 M HCl solution and further diluting to 1000 mL with double-distilled water [23]. The electrochemical experiments were carried out in a conventional three-electrode cell using a CHI 660B potentiostat/galvanostat (Shanghai Chenhua Instrumental Co., Ltd., China) at room temperature. An ITO glass substrate was used as the working electrode. The counter electrode and the reference electrode were platinum wire and saturated calomel electrode (SCE), respectively. The solutions were deaerated by a dry nitrogen stream and maintained with a slight overpressure of nitrogen during the experiments. A scanning electron microscope (SEM, S-4700, Japan) and X-ray diffraction (XRD, X' Pert-Pro MPD, PANalytical Company) were used to determine the morphology and the crystal structure of the sample nanomaterials, respectively.

### Preparation of the modified electrode

Before electrodeposition, ITO surface was ultrasonicated sequentially for 20 min in acetone, 10% KOH ethanol solution, and doubly distilled water. The electrodeposition process was conducted in a solution consisting of 1.0 mM H_2_PdCl_4 _and 0.33 M H_3_PO_4 _using cyclic voltammetry from -0.24 to 1.2 V with a scan rate of 50 mV s^-1^. The conventional Pd nanoparticles deposited on ITO were prepared by the potentiostatic method at a constant applied potential of -0.2 V in the solution as stated above. As-prepared Pd/ITO electrode was rinsed with water for three times and dried at room temperature. Before the activity test, the electrode was cycled at 50 mV s^-1 ^between -0.3 and 0.8 V in 0.5 M H_2_SO_4 _for at least 20 scans. After that the electrode was transferred to the cell containing 0.5 M H_2_SO_4 _+ 0.5 M HCOOH electrolyte solution. Subsequently, 20 scans were recorded at 50 mV s^-1 ^in the potential range -0.3 to 0.8 V. The amount of Pd (*W*_Pd_) loaded onto ITO was analyzed by an inductive coupled plasma emission spectrometer (ICP).

## Results and discussion

**Figure 1:**
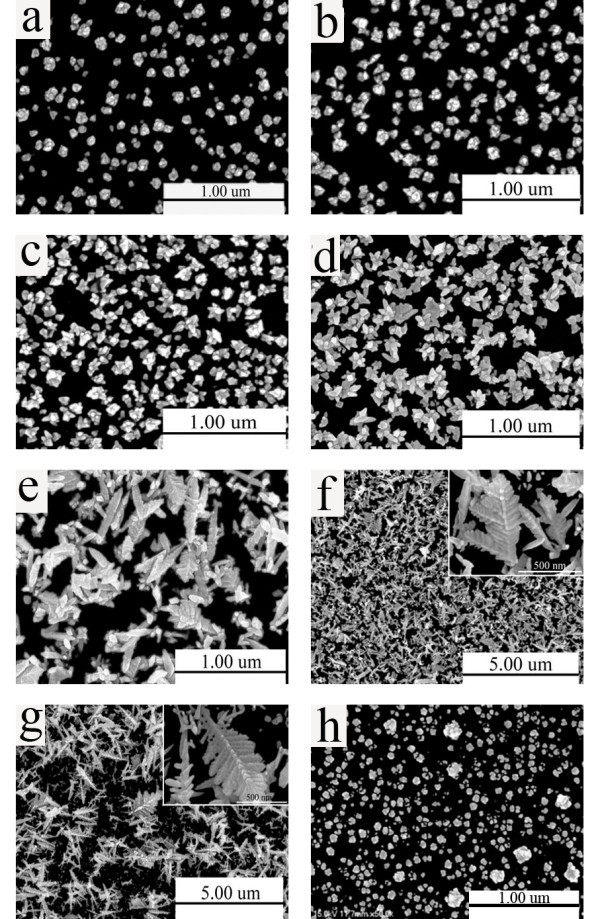
**SEM images of Pd nanostructures electrodeposited on ITO**. (1) Cyclic voltammetry deposition in 1.0 mM H_2_PdCl_4 _+ 0.33 M H_3_PO_4 _electrolyte for 5 cycles **(a)**, 10 cycles **(b)**, 20 cycles **(c)**, 35 cycles **(d)**, 75 cycles **(e)**, 100 cycles **(f)**, and 200 cycles **(g)**, and (2) potentiostatic deposition in 1.0 mM H_2_PdCl_4 _+ 0.33 M H_3_PO_4 _electrolyte **(h)**; inset is at a higher magnification.

Pine needle-like Pd micro/nano-leaves were prepared by a cyclic voltammetry method, i.e., electrodeposition in the presence of 1.0 mM H_2_PdCl_4 _+ 0.33 M H_3_PO_4 _electrolyte at room temperature. To observe the growth process of Pd micro/nano-leaves, as shown in Figure 1, the Pd nanoparticles were synthesized by controlling cyclic voltammetry electrodeposition from -0.24 to 1.2 V as a function of deposition cycles such as 5 (a), 10 (b), 20 (c), 35 (d), 75 (e), 100 (f), and 200 (g) cycles. At the initial stages (Figure 1a,b), featureless Pd nanoparticles of about 70 nm were formed. Extending the electrodeposition cycles, as shown in Figure 1c, d, Pd nanorod structure of 90 nm in width and 150 nm in length began to branch out. As the deposition cycles being further increased to 75 cycles, however, many nanoleaves started to form and grow from the edges of the nanorod particles, and a few completed nanoleaves with a short branch of 500 nm in length (Figure 1e) appeared. Further increasing the deposition cycles to 100 cycles, perfect Pd micro/nano-leaves were formed on the surface of ITO (Figure 1f). After 200 cycles, as shown in Figure 1g, the Pd micro/nano-leaves consisting of branches up to 500 nm in width and 1 μ m in length were formed, as shown in the high magnification image (inset in Figure 1g).

To pin down the related factors for the formation of Pd micro/nano-leaves, two control experiments have been carried out independently. First, replacing H_3_PO_4 _with other acids, e.g., H_2_SO_4_, HCl, HNO_3_, while keeping the other conditions unchanged, no Pd micro/nano-leaves were observed. It is proposed that the formation of Pd micro/nano-leaves is related to the effect driven by phosphate anions. Secondly, using a potentiostatic method instead of the cyclic voltammetry method and keeping the other conditions unchanged, featureless Pd nanoparticles (Figure 1h) were formed. Based on these observations, the existence of H_3_PO_4 _and the cyclic voltammetry method are two key factors, which are beneficial to the formation of Pd micro/nano-leaves. First, phosphate anions such as the hydrogen phosphate ion (HPO_4_^2-^) or the dihydrogen phosphate ion (H_2_PO_4_^-^) in solution are preferentially adsorbed on noble metal single crystals, which can greatly disturb the growth of the plane [24]. The phosphate anions are known to adsorb on the (111) surface of metal electrodes with a face-centered cubic (fcc) crystal structure. Especially, they have already been observed in the adsorption of both H_2_PO_4_^- ^and HPO_4_^2- ^on the Pt(111) [25]. Secondly, compared to the potentiostatic method, cyclic voltammetry is an alternating redox process, involving both electrodeposition and dissolution processes, which are critical to the formation of Pd nanoleaf structure. At the same time, varying the experimental conditions, such as the concentration, pH of the initial solution, reaction temperature, and time, may also effect the shape evolution [26].

**Figure 2:**
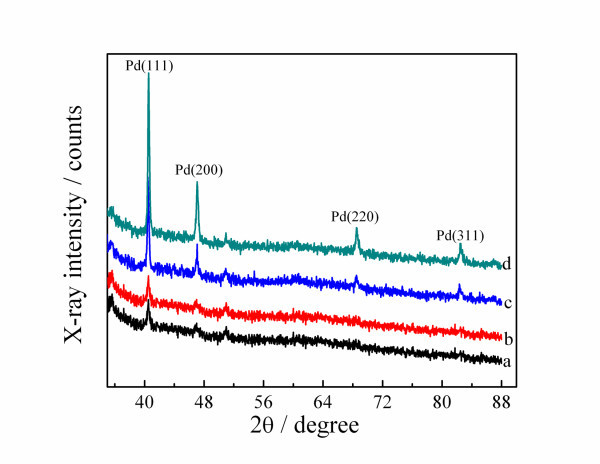
XRD patterns of Pt nanoparticles electrodeposited for 20 cycles **(a)**, 50 cycles **(b)**, 100 cycles **(c)**, 200 cycles **(d)**.

Figure 2 shows XRD patterns of Pd micro/nano-leaves prepared in the electrolyte consisting of H_2_PdCl_4 _and H_3_PO_4 _for 20 (a), 50 (b), 100 (c), and 200 (d) cycles. As seen from Figure 2, the impurity peak between 53° and 54° is attributed to the diffraction peak of SnO_2 _face (211), which is the main composition of the ITO glass. At the early stage, the well-defined peaks around 40° and 47° are observed and they are, respectively, attributed to the diffraction peaks of Pd crystal faces (111) and (200); as the cycles increase, the peaks around 68° and 83° appear, which could be indexed to the (220) and (311), respectively. All these demonstrate that Pd micro/nano-leaves possess an fcc structure.

**Figure 3:**
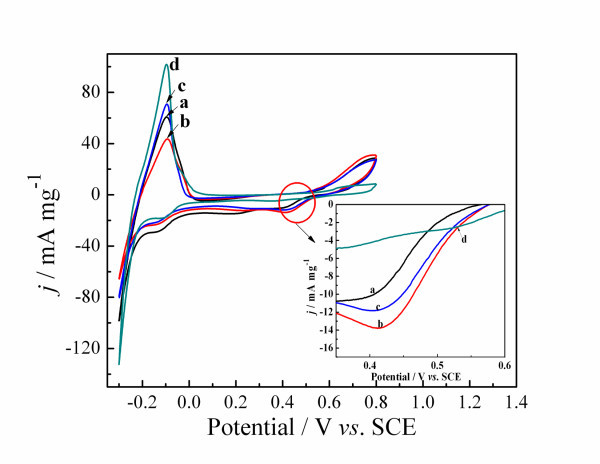
**CVs of Pd catalysts obtained from different deposition methods in 0.5 M H_2_SO_4 _solution**. (1) Cyclic voltammetry deposition for 20 cycles **(a)**, 35 cycles **(b)**, 100 cycles **(c) **and (2) potentiostatic deposition at -0.2 V **(d)**.

Inspired by their intriguing structure, Pd nanoparticles were tested as electrocatalysts. Figure 3 shows the cyclic voltammograms (CVs) of Pd nanoparticles recorded in a 0.5 M H_2_SO_4 _solution at 50 mV s^-1^. The shape of the profile is similar to what reported in literature [27]. The multiple peaks between -0.25 and 0 V are attributed to the adsorption and desorption of hydrogen. It is well known that the integrated intensity of hydrogen adsorption/desorption represents the number of available sites on catalyst [28]. It is also observed from Figure 3 that Pd electrodes produced by cyclic voltammetry deposition deliver reduction peaks at ca. 0.41 V while by potentiostatic deposition the reduction peaks shift to ca. 0.52 V. The peaks are attributed to the reduction of the oxide formed on the Pd during the forward scan. Compared to Pd nanoparticles, Pd micro/nano-leaves have the larger area of Pd oxide and lower reduction peak in the process of CVs. It is proved that Pd micro/nano-leaves have large active surface area and good electrocatalytic performance of as-prepared catalysts for the formic acid electro-oxidation.

**Figure 4:**
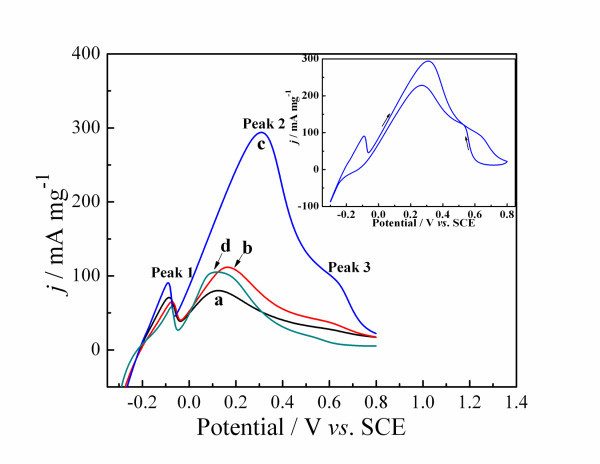
**CVs of Pd catalysts obtained from different deposition methods in 0.5 M HCOOH + 0.5 M H_2_SO_4 _solution at 50 mV s^-1^**. (1) Cyclic voltammetry deposition for 20 cycles **(a)**, 35 cycles **(b)**, 100 cycles **(c) **and (2) potentiostatic deposition at -0.2 V **(d)**.

The inset of Figure 4 shows the CV of formic acid oxidation on the Pd electrode, which was deposited for 100 cycles. In the forward scan, formic acid oxidation produced an anodic peak; while in the reverse scan, there was also an oxidation peak, which is attributed to formic acid oxidation after the reduction of the oxidized Pd oxide and the removal of the incompletely oxidized carbonaceous species formed in the forward scan. The oxidation peak in the forward scan is usually employed to evaluate the electrocatalytic activity of the catalysts and the anodic scan allows the formation and builds up of the poisonous intermediate, we thereby focus our observations on the evolution of the anodic scans, as is presented in Figure 4. From the curves shown in Figure 4, there are a main current peak between 0.1 and 0.4 V and two small current peaks near -0.1 and 0.6 V, respectively. The peak near -0.1 V is attributed to the adsorption and desorption of hydrogen, which is similar to that in Figure 3. The main peak between 0.1 and 0.4 V corresponds to formic acid oxidation via a direct pathway, while the peak near 0.6 V could be mainly attributed to formic acid oxidation via the CO pathway [29,30]. Moreover, the main peak is much larger than that near 0.6 V, indicating that the formic acid oxidation on Pd catalysts is mainly through the direct pathway. Especially in the curve a, b, and d, there are almost no peaks near 0.6 V. As observed from the curves a, b, c, and d in Figure 4, the onset potential of formic acid electro-oxidation locates near -0.04 V (a), -0.04 V (b), -0.07 V (c), and -0.05 V (d) vs. SCE, respectively, and the peak current density reaches 80.24 mA mg^-1 ^(a), 112.99 mA mg^-1 ^(b), 295.57 mA mg^-1 ^(c), 105.47 mA mg^-1 ^(d) for Pd catalysts, respectively. Among all the four electrodes, the Pd micro/nano-leaves exhibit the lowest onset potential and the highest current density of formic acid oxidation. This demonstrates that the electrocatalytic stability of the Pd micro/nano-leaves for formic acid oxidation is much higher than that of the Pd nanoparticles, which agrees with the literature [16]. Additionally, the commercial catalyst (E-TEK Pd/C) shows the peak current density at 190 mA mg^-1 ^in the same conditions (in a 0.5 M HCOOH + 0.5 M H_2_SO_4 _solution at 50 mV s^-1^) [31], which is lower than Pd micro/nano-leaves catalyst. Generally, catalytic performance of an electrode is assessed in CVs by the position and intensity of kinetically controlled process current on the potential scale. This may be attributed to the special structure that increases the electrochemically active surface area, thus greatly increases the activity for formic acid electro-oxidation.

## Conclusions

Using a simple electrodeposition method, Pd micro/nano-leaves were loaded onto a clean ITO. The Pd micro/nano-leaves are demonstrated to have superior performance in electrocatalytic activity toward the oxidation of formic acid.

## Abbreviations

CVs: cyclic voltammograms; fcc: face-centered cubic; ICP: inductive coupled plasma emission spectrometer; ITO: indium tin oxide; Pd: Palladium; SCE: saturated calomel electrode; SEM: scanning electron microscope; XRD: X-ray diffraction.

## Competing interests

The authors declare that they have no competing interests.

## Authors' contributions

RZ did the synthetic and characteristic job in this manuscript. WZ and HZ helped with the analysis of the mechanism for shape separation. YD is the PI of the project participating in the design of the study and revised the manuscript, and conducted coordination. PY, CW, and JX gave the advice and guide for the experimental section and edited the manuscript. All authors read and approved the final manuscript.
